# Sex-differences in the longitudinal recovery of neuromuscular function in COVID-19 associated acute respiratory distress syndrome survivors

**DOI:** 10.3389/fmed.2023.1185479

**Published:** 2023-06-26

**Authors:** Tea Lulic-Kuryllo, Marco Benedini, Marta Cogliati, Alessandro Cudicio, Bruno Guarneri, Stefano Gazzina, Simone Piva, Nicola Latronico, Claudio Orizio, Francesco Negro

**Affiliations:** ^1^Department of Clinical and Experimental Sciences, University of Brescia, Brescia, Italy; ^2^Department of Medical and Surgical Specialities, Radiological Sciences and Public Health, University of Brescia, Brescia, Lombardia, Italy; ^3^Neurophysiology Unit, ASST Spedali Civili University Hospital, Brescia, Lombardia, Italy; ^4^Department of Anesthesia, Critical Care and Emergency, ASST Spedali Civili University Hospital, Brescia, Italy; ^5^‘Alessandra Bono’ University Research Center on Long-Term Outcome (LOTO) in Critical Illness Survivors, University of Brescia, Brescia, Italy

**Keywords:** intensive care unit, motor unit, sex differeces, neuromuscular function, COVID-19

## Abstract

**Introduction:**

Patients admitted to the intensive care unit (ICU) following severe acute respiratory syndrome 2 (SARS-CoV-2) infection may have muscle weakness up to 1 year or more following ICU discharge. However, females show greater muscle weakness than males, indicating greater neuromuscular impairment. The objective of this work was to assess sex differences in longitudinal physical functioning following ICU discharge for SARS-CoV-2 infection.

**Methods:**

We performed longitudinal assessment of physical functioning in two groups: 14 participants (7 males, 7 females) in the 3-to-6 month and 28 participants (14 males, 14 females) in the 6-to-12 month group following ICU discharge and assessed differences between the sexes. We examined self-reported fatigue, physical functioning, compound muscle action potential (CMAP) amplitude, maximal strength, and the neural drive to the tibialis anterior muscle.

**Results:**

We found no sex differences in the assessed parameters in the 3-to-6-month follow-up, indicating significant weakness in both sexes.

Sex differences emerged in the 6-to-12-month follow-up. Specifically, females exhibited greater impairments in physical functioning, including lower strength, walking lower distances, and high neural input even 1 year following ICU-discharge.

**Discussion:**

Females infected by SARS-CoV-2 display significant impairments in functional recovery up to 1 year following ICU discharge. The effects of sex should be considered in post-COVID neurorehabilitation.

## 1. Introduction

Severe Acute Respiratory Syndrome 2 (SARS-CoV-2) has infected millions of individuals worldwide. SARS-CoV-2 infection negatively affects multiple organ systems, including the neuromuscular system (1, 2), especially in patients admitted to the intensive care unit (ICU). Patients admitted to the ICU due to SARS-CoV-2 infection present with muscle weakness for up to 1 year or more following ICU discharge (3–6), regardless of the disease severity. Sex differences in recovery of muscle function and fatigability were observed following ICU discharge in these patients. Specifically, females exhibit greater muscle weakness and fatigue up to 1 year following hospital discharge compared to males (3, 4). These findings suggest that the progression of recovery between the sexes following ICU discharge may be different, and rehabilitation protocols may need to be sex specific. The cause and mechanisms leading to greater muscle weakness in females are unclear, although muscle weakness may be a consequence of greater neuromuscular dysfunction. No studies to date have examined sex differences in neuromuscular function longitudinally following SARS-CoV-2 infection in patients discharged from the ICU. This knowledge is critical for post-COVID rehabilitation considering sex differences in disease outcome and progression were already established.

Patients infected with SARS-CoV-2 frequently present with neuromuscular dysfunction following infection. Other than muscle weakness, the neurophysiological tests commonly show low compound muscle action potential (CMAP) amplitude, polyphasic motor unit potentials, and spontaneous fibrillations (1). These findings suggest that motor unit neural control may be impaired, and only limited data are currently available (7). Since the increase in muscle force is modulated through the progressive recruitment of motor units and an increase in the motor unit firing rate, dysfunction in motor unit properties may lead to muscle weakness. Moreover, motor unit properties are different between healthy males and females in several leg muscles (8). For the tibialis anterior, a primary muscle involved in ankle dorsiflexion, healthy older females typically have lower motor unit firing rates than age-matched healthy males (9, 10), while overall leg strength is greater in males compared to females (10). Considering greater muscle weakness was demonstrated in females following ICU discharge for SARS-CoV-2 infection, it is likely that females have greater neuromuscular dysfunction than males following ICU discharge, possibly related to alteration in motor unit firing rate modulation. Furthermore, the literature lacks studies on the use of electromyographic (EMG), signal decomposition to monitor the progress of patients over time.

Therefore, the present study aimed to assess sex differences in longitudinal physical functioning following ICU discharge for SARS-CoV-2 infection. We hypothesized that females would display lower motor unit firing rates than males if no abnormalities in motor unit firing properties were present following ICU discharge. Further, we hypothesized that females would have greater muscle weakness than males post-ICU discharge. Lastly, we hypothesized that females would have lower CMAP amplitudes and greater physical impairment than males.

## 2. Materials and methods

### 2.1. Participants

This study was conducted on critically ill adult patients with confirmed SARS-CoV-2 infection admitted to the ICU at the Spedali Civili University Hospital in Brescia, Italy, from February 2020 to December 2021. All patients admitted to the ICU tested positive for SARS-CoV-2 infection on a C-reactive Protein test. The data presented in this study is part of a larger longitudinal study. The sample consisted of two groups of patients who were assessed longitudinally at either three- and six-months or six- and 12 months following ICU discharge. Specifically, seven females (64 ± 9.4 years) and seven males (64 ± 8.5 years) were assessed 3- and 6- months following ICU discharge, while 14 females (62 ± 8.8 years) and 14 males (62 ± 8.4 years) were assessed 6- and 12-months following ICU discharge. Patients were diagnosed with acute respiratory distress syndrome (ARDS) according to the Berlin criteria. Patients were invited to attend a post-ICU clinic, where the assessment was performed. Demographic information, including age, weight, and height of the participant was collected. This study was reviewed and received ethics approval from the Brescia Ethics Committee (NP3369) and conformed to the Declaration of Helsinki. Written informed consent was obtained from each participant prior to data collection.

### 2.2. Experimental design

Patients visited the post-ICU clinic on three occasions, where they performed several tests to examine their physical functioning, fatigue, peripheral nerve and muscle function, strength, and muscle activation. The experimental session started with an assessment of fatigue. Patients were asked to self-report activity limitations by filling out a short questionnaire that required patients to rate their fatigue level. This questionnaire provided a fatigue severity scale (FSS), with a higher score indicating greater fatigue. Following this, physical functioning was examined using the six-minute walking test (6MWT). The 6MWT is a standardized, objective assessment of physical performance, which tests both cardiopulmonary and skeletal muscle function. 6MWT was performed in accordance with the American Thoracic Society recommendations. The absolute distance walked in 6 min was measured.

**Figure 1:**
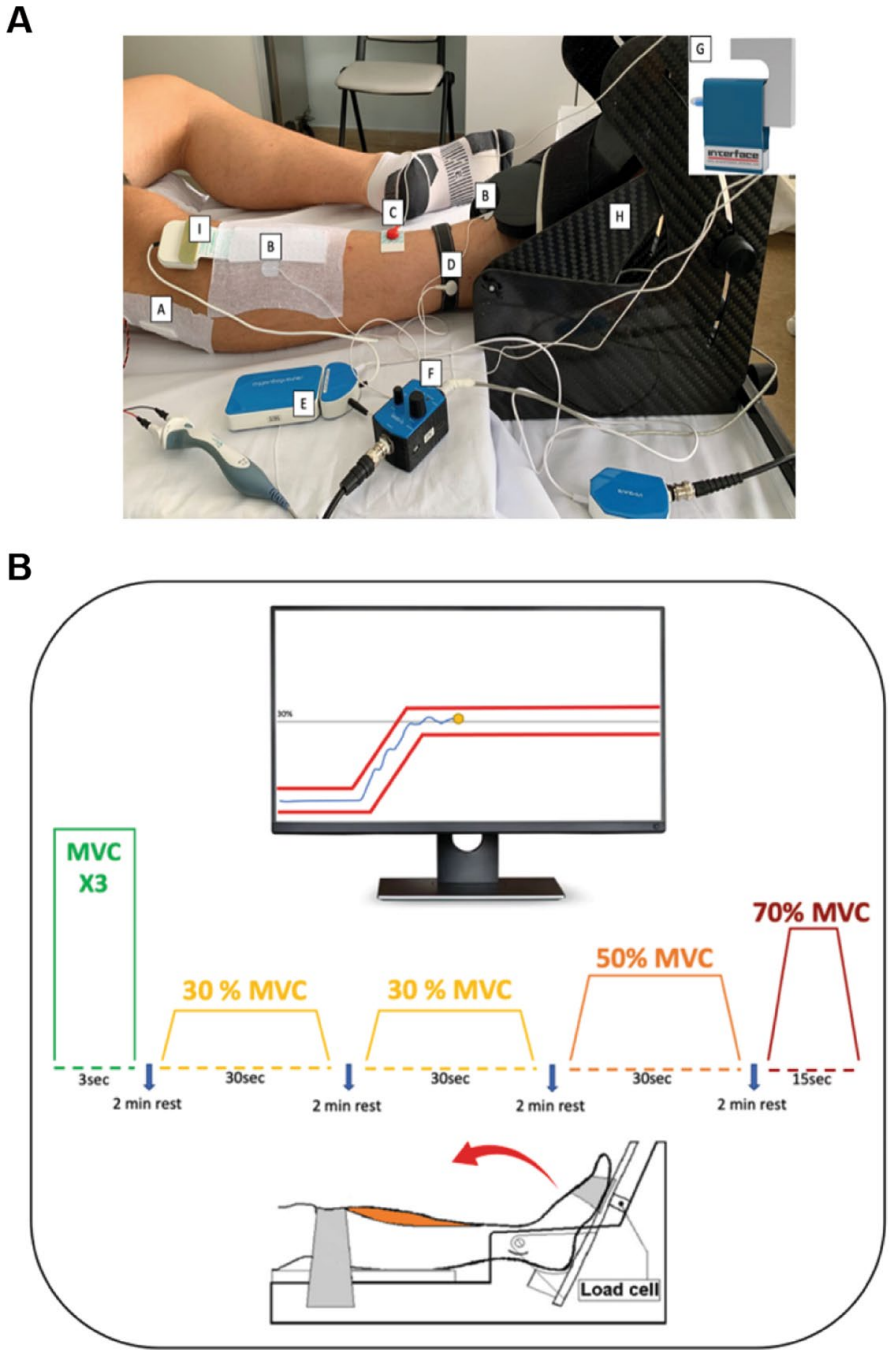
**A**: Protocol set-up: **(A)** Stimulator bar placed over the common peroneal nerve. **(B)** Surface electrodes for the identification of the CMAP-TA. **(C)** Stimulator ground electrode and **(D)** Ground electrode for the **(E)** EMG amplifier to prevent interference with biopotential signals. **(F)** Analog force amplifier to amplify the force signal detected by the **(G)** Load cell. The foot of the patient was strapped in a **(H)** carbon ankle-ergometer. **(I)** 64 Electrode Matrix was placed over the belly of the tibialis anterior muscle. **B**: Experimental protocol: With the help of the visual feedback, patients were asked to perform three maximal voluntary contractions (MVC) involving ankle dorsiflexion followed by submaximal trials at 30, 50 and 70% MVC. During the tasks at different MVC levels, the patients were required to keep the yellow dot between the red lines through ankle dorsiflexion.

Following 6MWT, patients were lying in bed, and the CMAP was used to assess the peroneal nerve function. CMAP was recorded using a novel technique with Nicolet Viking EDX (Natus Medical incorporated Middleton, WI). CMAP was obtained from the tibialis anterior muscle of the dominant leg using surface electrodes (Figure 1). The negative and positive electrodes were placed on the tibialis anterior muscle belly and distally on the tendon, respectively, with the ground electrode placed on the ankle of the same leg. The foot was strapped to a custom-made dynamometer equipped with a load cell (model SM-500 N). The knee was fully extended (180°), with the ankle in neutral position (110°) (11, 12). The common peroneal nerve was stimulated under the peroneal head with a bar electrode (BARR0026 - Spes Medica) with an interelectrode distance of 2.5 centimeters while patients remained fully relaxed. The stimulation started at 0 and automatically increased its intensity until the minimum stimulation intensity needed to elicit a maximal response from the tibialis anterior was identified. CMAP was defined as the action potential obtained at the maximal stimulation amplitude.

Following CMAP acquisition, patients performed maximal isometric ankle dorsiflexion with their dominant leg. Maximal voluntary contractions (MVC) were performed three times and involved maximal foot dorsiflexion with a 1.5-min rest between each trial. Each trial lasted 3 s to reach the MVC and 3 s of isometric contraction. Patients were verbally encouraged by the researchers throughout MVC performance. The peak force of three trials was used as a measure of MVC (11, 12). The load cell signals were amplified with an analog force amplifier (OT Bioelecttronica, Turin, Italy) connected to a portable bioelectrical signal amplifier (Sessantaquattro; OT Bioelettronica, Turin, Italy).

Following MVC performance, high-density surface electromyography (HD-sEMG) was applied to the tibialis anterior muscle (Figure 1). HD-sEMG signals were recorded in monopolar mode with an adhesive 64 electrodes matrix (GR08MM1305:13 rows by 5 columns, 8 mm IED; OT Bioelettronica, Turin, Italy). The matrix was placed following the guidelines of Barbero et al. (13) over the muscle belly thanks to a double-sided foam covered with conductive paste (NEURGEL250V–Spes Medica). Prior to applying the matrix, the skin was shaved and cleaned with abrasive paste (EVERI160SPE–Spes Medica) and water. Reference electrodes were positioned proximally over malleoli of the dominant leg. HD-sEMG signals were band-pass filtered (10–500 Hz), and sampled at 2000 Hz with a 16-bit A/D resolution.

Following rest, patients performed several submaximal isometric ramp-and-hold trials (Figure 1B). This task involved following a trapezoidal trajectory displayed on a computer monitor by contracting the tibialis anterior to generate torque using ankle dorsiflexion. Real-time visual feedback was provided to the patients throughout the trial. Patients performed submaximal trials at three levels: 30, 50, and 70% MVC randomly, with a 2-min rest between each trial. The 30% MVC trapezoid was repeated twice, and it was 36 s in duration, consisting of a 3-s ramp-up phase from baseline, a 30 s hold phase at 30% MVC, and a 3 s ramp-down phase. The 50% MVC trapezoid was 40 s in duration, consisting of a 5 s ramp-up phase from baseline, 30 s hold phase at 50% MVC, and a 5 s ramp-down phase. Lastly, the 70% MVC trapezoid was 29 s in duration, consisting of a 7 s ramp-up phase from baseline, 15 s hold phase at 70% MVC, and a 7 s ramp-down phase. All participants were familiarized with the experimental protocol prior to data collection.

### 2.3. Data analysis: motor unit decomposition

Monopolar HD-sEMG signals were decomposed into individual motor unit spike trains using a convolutive blind source separation algorithm previously validated (14). The EMG signals were band-pass filtered (3^rd^ order Butterworth, 20–500 Hz). The decomposition outputs were inspected by highly trained operators, and erroneous discharges were corrected. Only motor units with a silhouette value greater than 0.90 were used in further analyzes (14).

### 2.4. Data analysis: motor unit properties

Several motor unit properties were quantified from the decomposed data. First, mean firing rates and coefficient of variation for inter-spike-intervals were quantified from the hold phase of the trapezoidal submaximal contractions. For the 30% MVC, we analyzed the motor unit properties of low-threshold motor units, while for the 50% and 70% MVC, we examined only the high-threshold motor units. Recruitment and derecruitment thresholds (%MVC) were estimated from the generated torque of the first and last firing of the identified motor units. Firing rates at recruitment and derecruitment were quantified as the average of the first and last six firings of the identified motor units.

### 2.5. Statistical analyzes

All statistical analyzes were performed in SPSS (IBM, version 21). Data were checked for normality using the Shapiro–Wilk test. An independent two-sample t-test was used to assess age, weight, and height differences between groups. Most of the variables included in this study satisfied this condition. For the data that were normally distributed, a two-way repeated measures ANOVA was performed with a within-subject factor follow-up visit (Group 1: 3 and 6 months; Group 2: 6 and 12 months) and between-subject factor sex (Male, Female). Non-parametric statistical tests were used if data were not normally distributed. Specifically, to test group differences, the Mann–Whitney U test was used, while within-subject factor differences were tested using a Wilcoxon Signed Rank test. Significance was set to *p* < 0.05, and post-hoc tests were Bonferroni corrected.

### 2.6. Data availability

The data associated with the paper are available from the corresponding author upon request.

## 3. Results

Thirty-three patients were selected from a large number of subjects who took part in this study. Nine patients (4 females and 5 males) were assessed at 3-, 6- and 12- months and were allocated to both groups analyzed by the study, five patients (3 females and 2 males) were evaluated only at 3- and 6- months while 19 patients (10 females and 9 males) were evaluated at 6- and 12- months. It was not possible to assess all these patients in all three follow-ups due to the lockdown, which forcibly interrupted research activity in hospitals.

### 3.1. Patient demographics and physical functioning

Patient demographics and treatment details are presented in Table 1. There were no age differences between the sexes in patients assessed at 3- and 6-months (two samples t-test, *p* = 0.88) or 6- and 12-month follow-ups (two samples t-test, *p* = 0.82). There were no sex differences in the duration of hospitalization in our 3-to-6-month group (*p* = 0.93) nor 6-to-12-month group (*p* = 0.41). Similarly, no sex differences existed in the duration of mechanical ventilation in our 3-to-6-month group (*p* = 0.18) or 6-to-12-month group (*p* = 0.14). Additional data regarding corticosteroids and other drugs administered in ICU can be found in Table 1.

**Table 1:** Participant demographics with standard deviations.

	Group 1 (3–6 months)		Group 2 (6–12 months)	
	Female (*N* = 7)	Male (*N* = 7)	Female (*N* = 14)	Male (*N* = 14)
Age (years)	64 ± 9.4	64 ± 8.5	62 ± 8.8	62 ± 8.4
Height (cm)	163.1 ± 7	174.1 ± 9.3	161 ± 6.7	174.9 ± 8.1
Weight (kg)	84 ± 12.3	82.8 ± 13.4	77.7 ± 14.8	83.9 ± 12.3
Duration of Hospitalization Stay, days–Mean (SD)	28.7 ± 15.2	28 ± 16.1	34.1 ± 16.4	29.1 ± 15.4
Duration of ICU Stay, days–Mean (SD)	8.1 ± 5.7	9.4 ± 6.3	13.4 ± 10	9.9 ± 6.5
Intubation–*N* (%)	7 (100%)	5 (71.4%)	14 (100%)	13 (92.8%)
Duration of Intubation, days–Mean (SD)	8.4 ± 9	3.4 ± 2.7	12.4 ± 10.2	7.2 ± 5.9
ICU Catecholamine–*N* (%)	4 (57%)	1 (14.2%)	6 (42.9%)	6 (42.9%)
ICU Tocilizumab – N (%)	1 (14.2%)	0 (0%)	1 (7.1%)	3 (21.4%)
Steroids in ICU – N (%)	7 (100%)	6 (71.4%)	12 (86%)	13 (92.8%)
ECMO – N (%)	0 (0%)	0 (0%)	0 (0%)	0 (0%)
Tracheosotomy–*N*	1 (14.2%)	1 (14.2%)	4 (28.6%)	4 (28.6%)

ICU, Intensive Care Unit; ECMO, Extracorporeal Membrane Oxygenation.

### 3.2. Fatigue

No sex differences existed in fatigue scores at 3 or 6 months (3 months: *p* = 0.62; 6 months: *p* = 0.90; Table 2), nor at 6 or 12 months (6 months: *p* = 0.54; 12 months: *p* = 0.76; Table 3). However, in 3-to-6-month follow-up group, 2 out of 7 females and 3 out of 7 males had fatigue at 3 months, while 4 out of 7 females and 2 out of 7 males had fatigue at 6 months. Further, in 6-to-12-month follow-up group, 8 out of 14 females and 4 out of 14 males had fatigue at 6 months, while 6 out of 14 females and 3 out of 14 males had fatigue at 12 months.

**Table 2:** Summary of fatigue severity scale score, six-minute walking test score, and CMAP amplitude with standard deviations for participants tested at 3- and 6-months following ICU discharge.

	3 months		6 months	
	Female	Male	Female	Male
FSS	26.7 ± 18.1	30.1 ± 17.8	32.6 ± 20.6	30.5 ± 17.2
6MWT (m)	318.5 ± 95.9	381.4 ± 128.1	420 ± 99.4	490 ± 72.8
CMAP	6.3 ± 2.2	5.6 ± 1.3	7.4 ± 1.9	6.6 ± 1.3

FSS, fatigue severity scale; 6MWT, six-minute walking test; CMAP, compound muscle action potential.

**Table 3:** Summary of fatigue severity scale score, six-minute walking test score, and CMAP amplitude with standard deviations for participants tested at 6- and 12-months following ICU discharge.

	6 months		12 months	
	Female	Male	Female	Male
FSS	35.2 ± 18	30.5 ± 18.8	31.6 ± 20.1	27 ± 19.7
6MWT (m)	418.4 ± 132.7	506.4 ± 74.99	418.5 ± 115.6	510 ± 84.8
CMAP	7.3 ± 1.8	6.8 ± 1.4	7.9 ± 2	7.2 ± 1.9

FSS, fatigue severity scale; 6MWT, six-minute walking test; CMAP, compound muscle action potential.

### 3.3. Physical functioning

Patients assessed at 3- and 6-month follow-ups walked a 30% greater distance at 6 months (F1,12 = 14.2, *p* = 0.003, ηp^2^ = 0.5; Table 2). No sex differences (*p* = 0.17) and no interaction effects between sex and follow-up month (*p* = 0.90) were observed in this group. Males walked a greater distance than females in the 6- to 12-month follow-up group (F_1,24_ = 6.8, *p* = 0.01, η_p_^2^ = 0.22; Table 3). No differences in follow-up month (*p* = 0.55), and no interaction between sex and follow-up month (*p* = 0.98) existed.

### 3.4. Maximal torque

**Figure 2:**
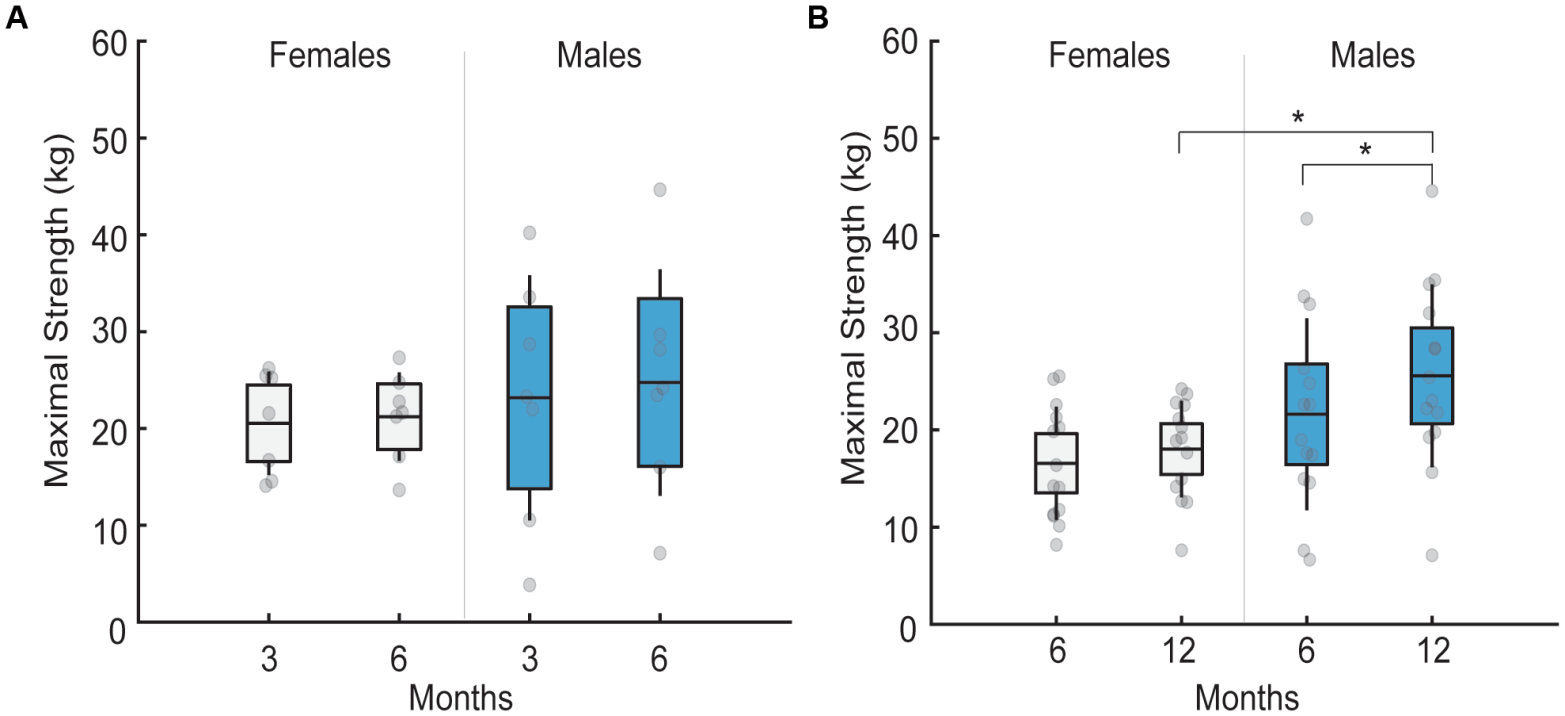
Mean maximal strength in males and females across different follow-up months. Black lines in the bar graphs denote means. Colored bars with lines denote 95% confidence intervals with standard errors. Scatter dots represent individual participant data. Brackets with stars denote significant differences between sexes or months. **(A)** Mean maximal strength for females (white) and males (blue) at 3 and 6 month follow-up. No sex differences existed across follow-up months. **(B)** Mean maximal strength for females (white) and males (blue) at 6 and 12 month follow-up. Males had greater maximal strength at 12 compared to 6 months. Males also had greater maximal strength than females at 12 months, but not at 6 month follow-up.

Maximal torque produced by the patients was similar between the sexes and follow-up visits in patients assessed at 3-to-6-month follow-up (*p* = 0.54; Figure 2A). In contrast, a significant interaction existed between follow-up month and sex for patients assessed at 6-to-12-month follow-up (F_1,26_ = 4.7, *p* = 0.03, η_p_^2^ = 0.15; Figure 2B). Specifically, at 12-months males had greater maximal torque than females (*p* = 0.013), but not at 6-months (*p* = 0.11). Maximal torque did not change in females (*p* = 0.059) but increased in males (*p* < 0.001) between 6 and 12-month follow-up visits.

### 3.5. Compound muscle action potential amplitude

CMAP amplitude was 17% greater at 6- compared to 3-months (F_1,12_ = 11.2, *p* = 0.006, η_p_^2^ = 0.48; Table 2) and 7% greater at 12- compared to 6-months (F_1,26_ = 5.06, *p* = 0.03, η_p_^2^ = 0.16; Table 3) irrespective of the sex. No sex differences existed in CMAP amplitude in 3-to-6-month follow-up (*p* = 0.41) or 6-to-12-month follow-up group (*p* = 0.42), and no interaction effects between sex and follow-up month were observed in either group (3-to-6-month follow-up: *p* = 0.76; 6-to-12-month follow-up: *p* = 0.61).

### 3.6. Motor unit properties

Due to technical difficulties, we were unable to decompose HD-sEMG signals in one female at 50% MVC and 70% MVC in our 3-to-6-month group and at 70% MVC in our 6-to-12-month group. Therefore, this data and their age-matched male data were not included in the analyzes.

### 3.7. Number of motor units decomposed

In our 3-to-6-month group, we decomposed a total of 202 and 209 motor units in females and 279 and 302 motor units in males at 30% MVC at 3 and 6 months, respectively. At 50% MVC, we decomposed a total of 31 and 43 motor units in females and 52 and 40 motor units in males at 3 and 6 months, respectively. Lastly, at 70% MVC, we decomposed a total of 44 and 48 motor units in females and 51 and 63 motor units in males at 3 and 6 months, respectively.

In our 6-to-12-month group, we decomposed 413 and 376 motor units in females and 611 and 532 motor units in males at 30% MVC at 6 and 12 months, respectively. At 50% MVC, we decomposed a total of 86 and 101 motor units in females and 124 and 166 motor units in males at 6 and 12 months, respectively. Lastly, at 70% MVC, we decomposed a total of 91 and 86 motor units in females and 151 and 163 motor units in males at 6 and 12 months, respectively.

### 3.8. Mean motor unit firing rates

**Figure 3:**
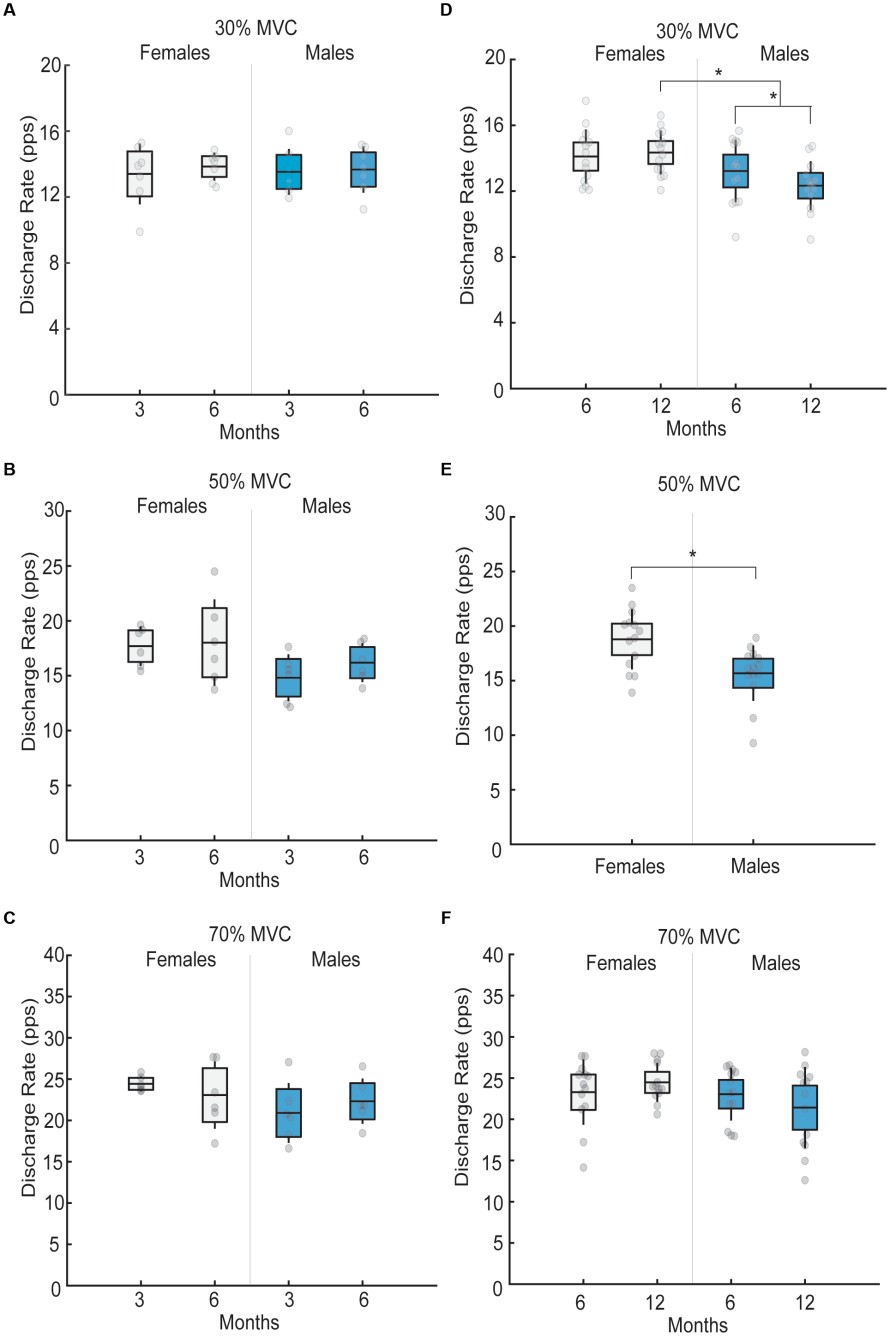
Mean motor unit firing rates in males and females across different follow-up months for 30, 50 and 70% submaximal tasks. Black lines in the bar graphs denote means. Colored bars with lines denote 95% confidence intervals with standard errors. Scatter dots represent individual participant data. Brackets with stars denote significant differences between sexes or months. **(A)** Mean motor unit firing rate for females (white) and males (blue) for 30% MVC at 3 and 6 month follow-up. No differences in mean motor unit firing rates were observed across months or sex. **(B)** Mean motor unit firing rate for females (white) and males (blue) for 50% MVC at 3 and 6 month follow-up. No differences in mean motor unit firing rates were observed across months or sex. **(C)** Mean motor unit firing rate for females (white) and males (blue) for 70% MVC at 3 and 6 month follow-up. No differences in mean motor unit firing rates were observed across months or sex. **(D)** Mean motor unit firing rate for females (white) and males (blue) for 30% MVC at 6 and 12 month follow-up. At 6-month follow-up, no sex differences in mean motor unit firing rates existed, but at a 12-month follow-up, mean motor unit firing rates were greater in females than males. Males also had lower mean motor unit firing rates at 12- compared to 6-months, while mean motor unit firing rates in females did not differ between the two visits. **(E)** Mean motor unit firing rate for females (white) and males (blue) for 50% MVC. Females had greater motor unit firing rates than males irrespective of the follow-up month (6 versus 12). **(F)** Mean motor unit firing rate for females (white) and males (blue) for 70% MVC at 6 and 12 month follow-up. No differences in mean motor unit firing rates were observed across months or sex.

In 3-to-6-month follow-up, no interaction effects between sex and follow-up month were observed in mean motor unit firing rate when patients performed isometric ramp-and-hold trials at 30% MVC (*p* = 0.79; Figure 3A), 50% MVC (*p* = 0.58; Figure 3B), and 70% MVC (*p* = 0.18; Figure 3C). Further, no sex differences were observed in the mean motor unit firing rate in this group at 30% MVC (*p* = 0.95), 50% MVC (*p* = 0.066), or 70% MVC (*p* = 0.18).

An interaction effect between sex and follow-up month was observed in mean motor unit firing rates in 6-to-12-month follow-up group. Specifically, at 30% MVC, a significant month by sex interaction existed (F_1,26_ = 4.70, *p* = 0.03, η_p_^2^ = 0.15; Figure 3D). At 6-month follow-up, no sex differences in low-threshold motor unit firing rates existed (*p* = 0.20), but at a 12-month follow-up, low-threshold motor unit firing rates were 2.01 pps greater in females than males (*p* < 0.001). Further, males had 0.89 pps lower low-threshold motor unit firing rates at 12- compared to 6-months (*p* = 0.006), while low-threshold motor unit firing rates in females did not differ between the two visits (*p* = 0.59). At 50% MVC, females had greater high-threshold motor unit firing rates than males, irrespective of the follow-up month (F_1,26_ = 9.5, *p* = 0.005, η_p_^2^ = 0.26; Figure 3E). Lastly, no differences in high-threshold motor unit firing rates between follow-up months and sexes existed at 70% MVC (all *p* > 0.05; Figure 3F).

### 3.9. Motor unit firing rate at recruitment

No month-by-sex interaction or main effects of sex existed in our 3- to 6-month follow-up or 6- to 12-month follow-up groups in firing rate at recruitment at any submaximal task level (all *p* > 0.05).

### 3.10. Motor unit recruitment threshold

No month-by-sex interaction or main effects of sex existed in our 3-to-6-month follow-up group in the recruitment threshold at any submaximal task level (all *p* > 0.05). In contrast, the recruitment threshold was greater at 12- compared to 6-month follow-up irrespective of the sex at 30% MVC (F_1,26_ = 5.6, *p* = 0.02, η_p_^2^ = 0.17), 50% MVC (F_1,26_ = 8.6, *p* = 0.007, η_p_^2^ = 0.24), and 70% MVC (Mann–Whitney U: *p* = 0.014).

### 3.11. Motor unit derecruitment threshold

Derecruitment threshold was not different between the sexes in 3- to 6-month follow-up at 30% MVC (Mann–Whitney U: all *p* > 0.05), 50% MVC (*p* = 0.47), or 70% MVC (*p* = 0.28). Similarly, no interactions between sex and follow-up month existed in the derecruitment threshold in 6- to 12-month follow-up group at 30% MVC (*p* = 0.97), 50% MVC (*p* = 0.95), or 70% MVC (*p* = 0.12).

## 4. Discussion

To our knowledge, this is the first study to objectively investigate sex differences in the longitudinal effects of SARS-CoV-2 hospitalization on physical functioning and neural drive to the muscle. Our findings revealed no sex differences in physical functioning, maximal torque, fatigue, CMAP amplitude, or motor unit properties in the 3-to-6-month follow-up group. In contrast, sex differences existed in physical functioning, maximal torque, and motor unit firing characteristics but not fatigue or CMAP amplitude in our 6-to-12-month follow-up group. Our findings demonstrate that neuromuscular function is impaired in both males and females, up to 6 months following ICU-discharge for SARS-CoV-2 following which recovery at 1 year is only observed in males. Additionally, to our knowledge, this is also the first study to longitudinally evaluate changes in neural drive to muscles in a disease state.

### 4.1. Impairments in physical functioning

Physical impairment was present in our 3-to-6-month follow-up group in both, males and females. In healthy adults, males typically walk a greater distance than females when not corrected for height (15). However, we did not observe these differences in our data. Both, males and females, had impairments in physical functioning at 3-months following ICU discharge as their mean scores for the 6MWT fell well below the typical scores for healthy older adults (15, 16). Moreover, the 6-min walking distance at 3 months in females was lower compared to patients who had the classic ARDS (females: 318 meters vs. ARDS: ~360 meters) (17) and SARS-CoV-2 patients assessed at 3 months (382 meters) (18). These findings align with previous observations that female ARDS patients typically have lower 6MWT results (17). In contrast, male six-minute walking distance at 3 months was slightly less impaired than that of ARDS patients (males: 381 meters vs. ARDS: ~360 meters), similar to that of SARS-CoV-2 patients tested at 3-months following infection (382 meters) (18), but below those values achieved in healthy older males (healthy older males: ~690 meters) (16). These results indicate that males affected by and hospitalized for SARS-CoV-2 may be less impaired than classic ARDS patients, but still have substantial physical impairments compared to healthy older males. By 6-months, both sexes exhibit substantial increases in six-minute walking distance and have less impairment than classic ARDS patients [for reference values, see Parry et al. (17)] although the values for both sexes still fall below those of healthy older adults.

In our 6-to-12-month follow-up group, males walked a greater distance than females irrespective of the follow-up month, which aligns with what is typically observed in healthy adults (15, 16, 19). However, female results still fell below those reported in classic ARDS patients (17) and were significantly below those documented in healthy older females (16). In contrast, males showed less impairment than classic ARDS patients, but their results still fell below those observed in healthy older males. Moreover, observed sex differences in healthy older adults are typically between 30 to 62 meters (16, 19). However, in our study, this difference was ~88 meters at 6 months and 91 meters at 12 months, indicating that female recovery in physical functioning is lagging. Females admitted to the ICU usually have greater illness severity (20), which may contribute to reductions in peak exercise aerobic capacity (21). This is partly due to the deconditioning and muscular limitation. Therefore, greater impairments in the cardiopulmonary or muscular system may be the contributing factor to the impairments in physical functioning observed in females.

### 4.2. Strength impairments

Healthy older males have greater strength than females in ankle dorsiflexion (10). The absence of sex differences in strength in our 3-to-6-month follow-up group and at 6-months in our 6-to-12-month follow-up group indicates the presence of muscle weakness in males, although muscle weakness in females cannot be ruled out. By 12-months, sex differences in maximal strength are observed and the differences can be primarily attributed to the increase in muscle strength in males, which reaches similar values to that reported in healthy older adults (11). These findings indicate that males recover their maximal strength by 12-months following ICU-discharge. Considering that muscle strength does not change in females from 3 to 6 months nor 6 to 12 months, these data suggest that females continue to exhibit muscle weakness up to 1 year following ICU discharge. These findings support those of Huang et al. (4), which found greater muscle weakness in females 6- and 12-months post ICU-discharge compared to males. Patients requiring hospitalization and ICU stay are at greater risk of muscle atrophy, sensory-motor axonal polyneuropathy (critical illness polyneuropathy) and myopathy (22) due to pathological changes such as increased inflammation, mitochondrial dysfunction, and reduced physical activity. Females have a more than four fold greater risk of developing ICU acquired weakness during the ICU stay than males (23, 24) and thus, longitudinal muscle weakness in females may be a consequence of multiple factors including polyneuropathy, myopathy, and/or greater muscle atrophy (22, 23). Additionally, corticosteroid therapy during ICU was also shown to be associated with greater muscle weakness at 12 months (4).

### 4.3. Altered motor unit firing

The central nervous system controls muscle force production by modulating the number of recruited motor units and their firing rate. In healthy older adults, sex differences exist in tibialis anterior motor unit firing rates, such that females have lower motor unit firing rates than males when submaximal isometric torques are generated at minimal [25% MVC (10)] to high force levels [100% MVC (9)]. Despite this, we found no sex differences in motor unit firing rates in our 3-to-6-month follow-up group. Moreover, we found no sex differences in low-threshold motor unit firing rates at 6 months post-ICU discharge in our 6-to-12-month follow-up group. These findings indicate that patients discharged from the ICU following SARS-CoV-2 infection have impaired low-threshold motor unit firing rates up to 6 months following ICU discharge. Further, sex differences in low-threshold motor unit firing rates were present at 12-months following ICU discharge. These differences were due to a decrease in motor unit firing rate in males from 6 to 12-month follow-up. In contrast, female low-threshold motor unit firing rates remained the same across follow-up months. Moreover, we also observed greater high-threshold motor unit firing rates in females in our 6-to-12-month follow-up group, irrespective of the follow-up month. These findings indicate that motor unit firing rates in males are elevated up to 6 months following ICU discharge and decrease with recovery by 1 year. Decrease in motor unit firing rates in our patients occurred concurrently with improvements in strength, physical functioning, and CMAP amplitude in males.

These findings demonstrate that patients hospitalized for SARS-CoV-2 infection require increased neural input to optimize muscle force production in the first 6-months following ICU discharge and agree with previous observations in patients with myopathic disease (25). As the muscle recovers, there is less requirement for increased neural input to the muscle. Thus, neuromuscular system becomes more efficient at force production 1 year following ICU discharge in males, signifying neuromuscular recovery. In contrast, females do not recover in terms of physical functioning and strength, and thus, the neural input to the muscle remains high even 1 year following ICU-discharge. The exact mechanisms associated with the lack of recovery in motor unit firing rates in females are unclear but may be due to structural changes in the muscle fiber, which consequently affect motor unit firing rates. Considering that females tend to lose significantly more muscle mass than males in the ICU, it is likely that some motor unit denervation occurs as a consequence of concurrent critical illness polyneuropathy. Due to motor unit loss, motor units must fire at higher rates to produce a desired force at low force levels. Moreover, patients with myopathy commonly exhibit high motor unit firing rates (26), which may explain the current findings in females.

### 4.4. Lack of sex differences in CMAP amplitude, fatigue, and other motor unit characteristics

Our study demonstrates that there is a significant recovery of CMAP amplitudes at 1 year following ICU discharge for SARS-CoV-2 infection, irrespective of sex. A previous study did not find reductions in peroneal nerve CMAP amplitude in SARS-CoV-2 patients (27). However, none of their patients required ICU treatment, and half did not require hospitalization, which may have resulted in differences between our findings and theirs. Reduced peroneal and tibial nerve CMAP amplitude in ICU admitted SARS-CoV-2 patients were documented previously in case studies (28, 29). Reduced CMAP amplitudes are observed in patients who have critical illness myopathy and critical illness polyneuropathy (30), which cannot be discounted in our patient group.

We observed no differences in fatigue scores between the sexes in either group, although a larger proportion of females reported fatigue compared to males. Fatigue is a commonly reported symptom in SARS-CoV-2 patients (31), and it is more prominent in females (32).

Lastly, we observed an increase in recruitment threshold of low and high-threshold motor units between 6- and 12-months following ICU discharge irrespective of the sex while no differences in recruitment threshold between months or sexes were observed in our 3-to-6-month follow-up group. This increase in recruitment threshold force indicates that the identified motor units were recruited later during the isometric contraction. In general, due to the bias of the surface decomposition algorithms toward higher threshold units (33, 34), it is not straightforward to link this result to an underlying neurophysiological mechanism. Probably, due to tendency to have higher MVC and, therefore, likely, higher motor unit recruitment after 6 months, the complexity of the EMG signal was increasing during the follow-up resulting in the tendency for the decomposition algorithm to identify higher threshold units.

### 4.5. Limitations

Our study has several limitations. First, the sample size in our 3-to-6-month follow-up group is small and should be considered when interpreting the findings. In general, females are less likely to be admitted to the ICU (20) and ARDS is more frequent in males than females (35), making the recruitment of female patients challenging. Assessment at all time points for all patients in this study was not possible due to patient’s unwillingness to participate longer than 6 months or late recruitment of the patients at 6 months. Second, we did not recruit a control group for this study as the likelihood of asymptomatic SARS-CoV-2 infection is high in the general population, making it difficult to control for the lack or history of infection. Even mild SARS-CoV-2 infection was shown to negatively affect the neuromuscular system in individuals who were not hospitalized (27). We did not examine structural adaptations to the muscle, including atrophy, which may have contributed to muscle weakness.

## 5. Conclusion

In conclusion, we demonstrated sex differences in longitudinal neuromuscular function in patients infected with SARS-CoV-2 following ICU discharge, specifically in physical functioning, maximal strength, and motor unit firing rates. Our data demonstrated that females display significant impairments in functional recovery up to 1 year following ICU discharge for SARS-CoV-2. Therefore, the effects of sex should be considered in post-COVID neurorehabilitation.

## Data availability statement

The raw data supporting the conclusions of this article will be made available by the authors, without undue reservation.

## Ethics statement

The studies involving human participants were reviewed and approved by Brescia Ethics Committee. The patients/participants provided their written informed consent to participate in this study.

## Author contributions

TL-K, MB, and MC: conceptualization, methodology, investigation, formal analysis, writing original draft, writing review and editing, and visualization. AC: conceptualization, methodology, investigation, writing original draft, writing review and editing, and visualization. BG and SG: investigation, visualization, and writing review and editing. SP, NL, CO, and FN: conceptualization, methodology, writing original draft, writing review and editing, visualization. All authors contributed to the article and approved the submitted version.

## Funding

The study was funded by the Italian Ministry of University and Research, project COVI19-FATIGUE, Ref. FISR 2020 COVID: FISR2020IP_01339. Marco Benedini’s PhD scholarship was partially funded by Rotary Club Brescia Nord.

## Conflict of interest

The authors declare that the research was conducted in the absence of any commercial or financial relationships that could be construed as a potential conflict of interest.

## Publisher’s note

All claims expressed in this article are solely those of the authors and do not necessarily represent those of their affiliated organizations, or those of the publisher, the editors and the reviewers. Any product that may be evaluated in this article, or claim that may be made by its manufacturer, is not guaranteed or endorsed by the publisher.
